# Impact of climate change on food systems – the One Health approach

**DOI:** 10.1093/eurpub/ckac129.019

**Published:** 2022-10-25

**Authors:** R Assunção

**Affiliations:** 1 IUEM, Instituto Universitário Egas Moniz, Caparica, Portugal; 2 Centre for Environmental Sciences and Marine, Aveiro, Portugal

## Abstract

In recent decades, changes in climate have caused impacts on natural and human systems on all continents and across the oceans. Climate change has become one of the most critical issues for the sustainable development of human societies and the functioning of ecosystems on Earth. In one hand, climate change threatens our ability to ensure global food security, eradicate poverty and achieve sustainable development. Agriculture and animal production are affected by changing rainfall patterns, drought, flooding and the geographical redistribution of pests and diseases, with consequent implications in the food availability, a key requirement for food security. On the other hand, despite less debated, climate change could also affect food safety, impacting the occurrence of food safety hazards at various stages of food chain, from “farm to fork”. The tendency to increase the use of agrochemicals to balance the effects of more frequent extreme weather events and water scarcity in some regions could become more frequent. In addition to pesticide residues, both chemical and microbiological risks are expected to impair food and feed safety as a consequence of climate change: in particular mycotoxins, marine biotoxins (phycotoxins), trace metals, among others. Humans, animals and the environment are/will be affected by the consequences of climate change, with an expected impact on the food systems. Thus, a One Health perspective, representing a holistic view of the problems, defining and establishing adequate strategies to tackle these challenges, is more than needed. On this presentation, main issues relating the impact of climate change on health of humans, animals and environment and how a One Health perspective, as a holistic approach, represent a key contribution to the definition of proper policies to ensure the public health will be approached and debated.

